# Canine leishmaniosis and peripheral neuropathy: a lesson from the neurologist

**DOI:** 10.1186/s13071-025-06773-4

**Published:** 2025-06-11

**Authors:** Floriana Gernone, Domenico Otranto, Jairo Alfonso Mendoza-Roldan, Annamaria Uva, Kaspar Matiasek, Andrea Zatelli

**Affiliations:** 1 Department of Veterinary Medicine, University of Bari Aldo Moro, Bari, Italy; 2 Department of Veterinary Clinical Sciences, Jockey Club College of Veterinary Medicine and Life Sciences, City University of Hong Kong, Hong Kong, China; 3 Section of Clinical and Comparative Neuropathology, Ludwig-Maximilians-Universität, Munich, Germany

**Keywords:** *Leishmania infantum*, Neurological signs, Peripheral nervous system, Blood–nerve barrier, Pathogenesis, Dogs

## Abstract

**Background:**

Canine leishmaniosis (CanL), a sand fly-borne zoonotic disease caused by *Leishmania infantum*, is potentially lethal in dogs. A similar or slightly higher quantity of antigens over antibodies promotes the formation of soluble circulating immune complexes (sCIC), which are deposited in the capillary wall, causing the inflammatory cascade responsible for clinical manifestations. Nervous system involvement during CanL is rarely reported in both humans and dogs, and the exact underlying process involving the peripheral nervous system (PNS) is still debated in both species.

**Methods:**

Two male mixed-breed dogs were presented for exercise intolerance, non-ambulatory flaccid tetraparesis and decreased/absent flexor reflexes in all four limbs. Both dogs were seropositive for *L. infantum* and presented clinicopathological abnormalities suggestive of active CanL. One dog had received *N*-methyl-glucamine antimoniate two months before presentation without neurological improvement.

**Results:**

Generalized PNS involvement was confirmed in both dogs. Biopsies of muscle and nerve tissues showed mononuclear cell inflammatory infiltration, and quantitative real-time polymerase chain reaction (PCR) was positive for *Leishmania* spp. In addition, *Leishmania* spp. antigen was detected in the nerve from one dog by immunohistochemistry. Both dogs were started on *N*-methyl-glucamine antimoniate and allopurinol in association with immunosuppressive corticosteroid therapy, recovering in few weeks.

**Conclusions:**

Peripheral neuropathies during active CanL can be a consequence of sCIC deposition on endoneurial vascular endothelium comprising the blood–nerve barrier and its consequent breakdown. However, an abnormal host immune response triggered by *L. infantum* causing demyelination and/or axonal disruption is also possible. The positive response to the immunosuppressive therapy further supports an immune-mediated origin of the PNS condition. Therefore, CanL should be included in the differential diagnosis of PNS disease in dogs, especially in areas endemic for *L. infantum*.

**Graphical Abstract:**

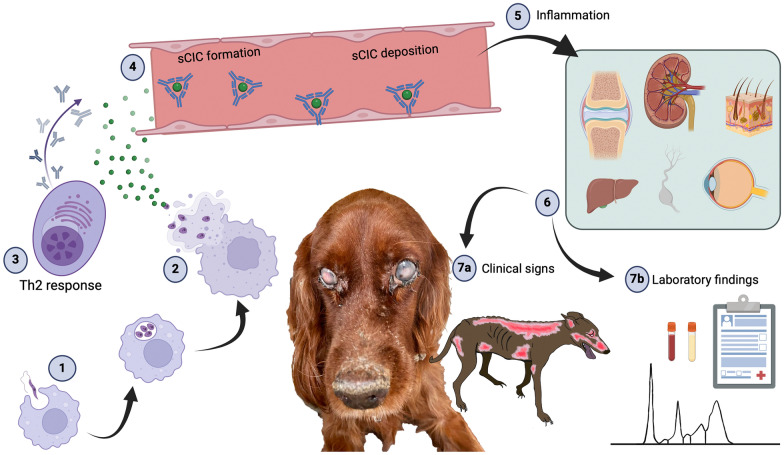

## Background

**Fig. 1:**
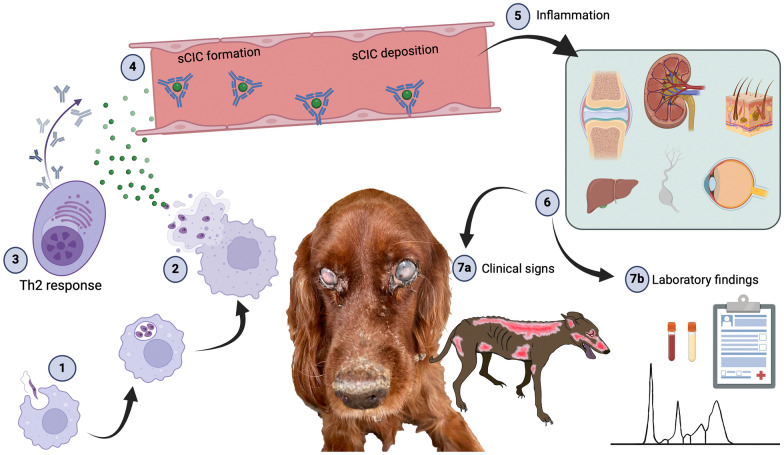
Pathogenetic mechanism during canine leishmaniosis. During a blood-feeding by the sand fly, the promastigotes enter the cytoplasm of the host macrophages (1), and lose the flagellum, becoming amastigotes. Inside the cytoplasm of the macrophages, the parasites replicate, causing cellular membrane rupture leading to the release of other amastigotes (2). Activation of the Th2 response by the host leads to the formation of antibodies (3) which, if present at levels similar to the antigens, lead to the formation of soluble circulating immune complexes (sCIC) (4). Soluble circulating immune complexes diffuse in the bloodstream and are deposited in the capillary beds (4), causing a cascade of events (e.g. the fixation of complement, recruitment of neutrophils, vascular wall damage and the localized leakage of fluid, proteins and inflammatory cells) (5). The deposition of sCIC occurs in predilected tissue (e.g., renal glomerulus, the anterior uvea, dermis, nasal mucosa, synovial membrane and choroid plexus) supplied by vessels with specific characteristics such as high pressure, turbulent flow and small diameter (6). Consequently, the deposition of immune complexes at these sites contributes to the pathophysiology and to the clinical (7a) and clinicopathological (7b) findings

Canine leishmaniosis (CanL) is a sand fly-borne zoonotic disease caused by *Leishmania infantum* (Kinetoplastida, Trypanosomatidae) [1–3], which may be lethal in dogs. The disease progression and the clinical outcome are dependent on several factors, such as the individual host immune response [4, 5], with lymphocyte T helper 2 (Th2) humoral response leading to development of clinical signs due the deposition of soluble circulating immune complexes (sCIC) [5] (Fig. 1). A similar or a slightly higher quantity of antigens over antibodies promotes the formation of sCIC, which deposit in the capillary walls [6]. The deposition of sCIC is contingent upon several factors, including the nature of the antigen, the characteristics of antibodies, the vascular wall permeability and the endothelial damage [8]. High pressure and turbulent flow favour the deposition of sCIC within the vessel wall [9]. In this scenario, the deposition of sCIC promotes a cascade of events, including the fixation of complement, recruitment of neutrophils, vascular wall damage and the localized leakage of fluid, proteins and inflammatory cells [4]. Deposition of sCIC tends to occur in specific organs [7], such as the renal glomerulus, the anterior uvea, dermis, nasal mucosa, synovial membrane and choroid plexus [10, 11], causing the typical clinical manifestations (Fig. 1) of CanL including generalized lymph node enlargement, desquamative and ulcerative dermatitis, vasculitis, glomerulonephritis, uveitis, arthritis/polyarthritis and meningitis [8, 12–15]. Despite its clinical spectrum, neurological manifestations during CanL are rarely (i.e. case reports or case series) described [11–33], with an unclear pathogenetic mechanism [34, 35]. A number of neurological clinical signs (e.g. seizures, paresis/paralysis, ataxia, generalized weakness, muscle atrophy) have been reported in the course of CanL as a consequence of the central and peripheral nervous system (PNS) involvement [15–33]. Additionally, histopathological findings (e.g. perivascular lymphoplasmacytic infiltrates, signs of neuronal degeneration, diffuse gliosis, satellitosis, leptomeningitis, choroiditis, vascular congestion and areas of focal microhaemorrhages) have been described in *L. infantum*-seropositive dogs, even without neurological signs [20, 36]. On the other hand, in human medicine, there is evidence of neurological involvement in patients with visceral and cutaneous leishmaniasis, with the following clinical classification: (a) peripheral demyelinating immune-mediated neuropathy in visceral leishmaniasis, (b) peripheral neuropathy in cutaneous leishmaniasis by direct or close parasite involvement with the nerve or nerve sheath and (c) central nervous involvement by haematogenous dissemination [37]. The aims of the present manuscript are to describe PNS alterations in two leishmaniotic dogs and to discuss the potential pathogenetic mechanism responsible for the neurological signs.

## Methods

### Case 1

A 3-year-old male mixed-breed neutered dog, living in an endemic area for CanL in southern Italy [38], was referred because of progressive non-ambulatory flaccid tetraparesis over the last 3 months.

Two months prior to presentation, the dog had been treated, without improvement in neurological signs, with *N*-methyl-glucamine antimoniate (100 mg/kg/day) for 4 weeks, following a positive indirect immunofluorescence test (antibody titre 1:320) for *L. infantum*.

### Case 2

A 2-year-old mixed-breed male neutered dog, living in an endemic area for CanL in southern Italy [38], was referred because of progressive and insidious exercise intolerance and weakness over a few days.

## Results

### Case 1

**Fig. 2:**
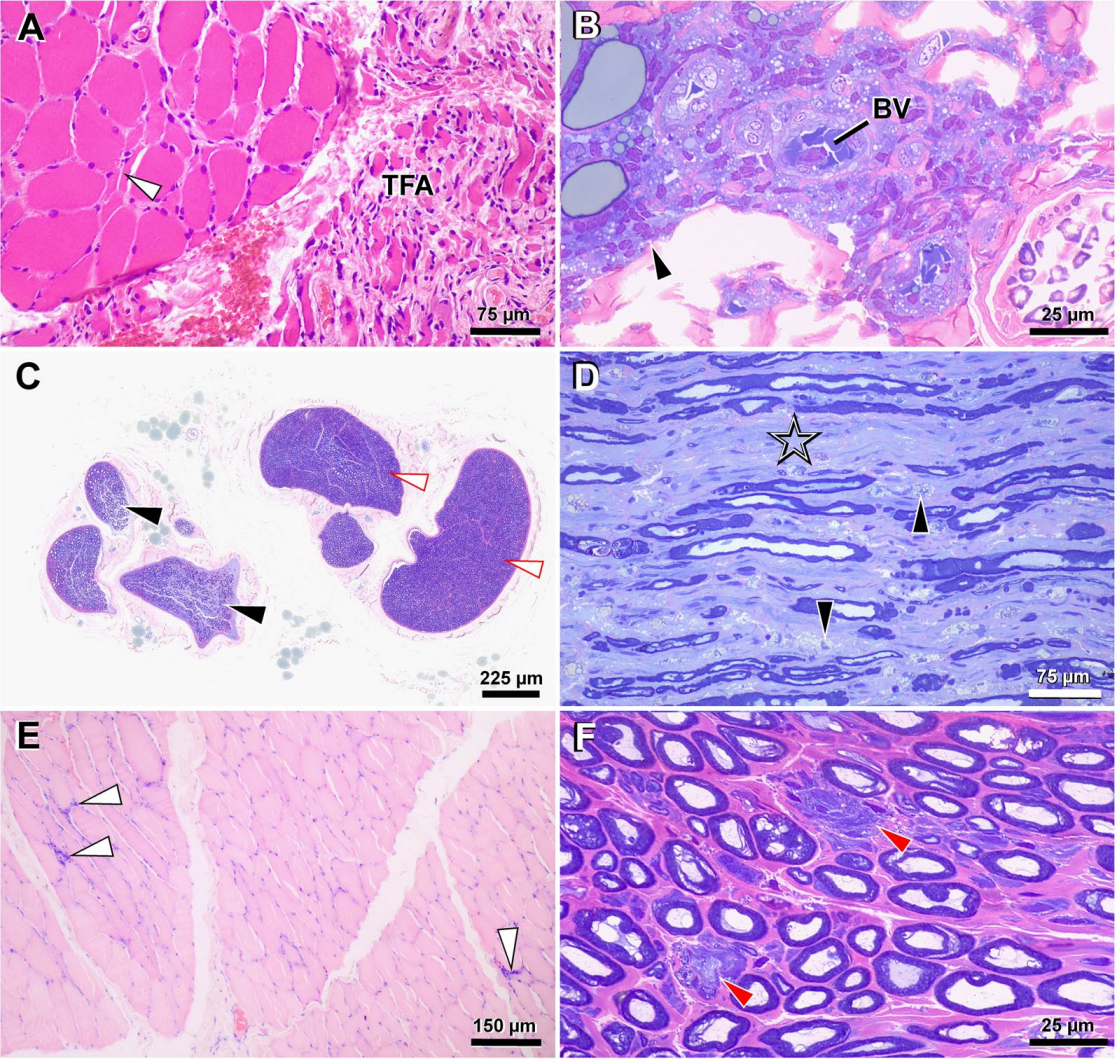
Muscle and nerve biopsy findings in case 1 (**A**–**D**) and 2 (**E**, **F**). In case 1, the tibialis cranialis muscle biopsy predominantly featured a denervation pattern with acute angular fibre atrophy (**A**: white arrowhead) next to entire fascicles undergoing total fibre atrophy (**A**: TFA). Only in the epifascial connective tissue, there were several inflammatory foci characterized by perivascular infiltrates by predominantly histiocytes and macrophages (**B**: black arrowhead). The common peroneal nerve presents with a typical distribution pattern of an inflammatory neuropathy of multiplex type (**C**). Individual fascicles show focal myelinated fibre losses (**C**: black arrowheads), while others remain histologically unremarkable (**E**: red arrowheads). In the power field, there are large gaps in between myelinated fibres (**D**: asterisk), and multiple macrophages are seen within the endoneurium (**D**: black arrowheads). Case 2 showed mild interstitial inflammatory foci in the gastrocnemius muscle (**E**: white arrowheads). Common peroneal nerve sections showed multiple fibres undergoing chronic inflammatory demyelination (**F**: red arrowheads). Magnification: see scale bars. Embedding and stains: **A**: paraffin, haematoxylin–eosin; **B**, **C**, **D**, **F**: epoxy resin, toluidine blue-safranin O; **E**: paraffin, Giemsa. Abbreviations: **A**: TFA: total fibre atrophy; **B**: BV: blood vessel

No abnormalities were noted on physical examination besides the neurological signs suggestive of generalized PNS involvement (i.e. head ventroflexion, non-ambulatory flaccid tetraparesis, no proprioceptive deficits in all four limbs, absence of flexor reflexes in all four limbs, generalized muscle atrophy). Because of suspicion of inflammatory/immune-mediated/infectious, neoplastic or degenerative disease, complete blood and urine tests were performed. The main clinicopathological findings were increased total serum protein [7.8 g/dl (5.7–7.3) with decreased albumin/globulin ratio [0.62 (0.7–1.30)], increased globulins [4.8 g/dl (2.8–3.9)], hypergammaglobulinemia [17.9% (6.6–14.5%)], and increased acute-phase proteins such as C-reactive protein [0.56 mg/l (0.01–0.45)] and serum ferritin [721 μg/l (80–270)]. The clinicopathological findings associated with positivity for anti-*L. infantum* antibodies (16.8%) by enzyme-linked immunosorbent assay [37, 39]. This was also confirmed by the detection of *Leishmania* spp. amastigote in bone marrow cytology. Electrodiagnostic testing was declined by the owner, who instead agreed to perform muscle and nerve biopsies. Muscle biopsy of the left tibialis cranialis femoris showed marked, multifocal, panfascicular and chronic muscle atrophy as a consequence of denervation (Fig. 2A–D). Several inflammatory foci were detected in the epifascial connective tissue, characterized by perivascular infiltrates of predominantly histiocytes and macrophages (Fig. 2A, B). Preparations of the left common peroneal nerve showed a pronounced chronic multifocal demyelinating inflammatory neuropathy involving large fibres with a mild endoneurial mononuclear infiltration (Fig. 2C, D). The histopathological findings were consistent with an immune-mediated neuropathy. In addition, *Leishmania* spp. antigen was identified by immunohistochemistry of the nerve specimen. Based on the clinical and histopathological findings, the dog was started on *N*-methyl-glucamine antimoniate (100 mg/kg daily for 4 weeks) and allopurinol (10 mg/kg daily for 6 months) in combination with immunosuppressive corticosteroid therapy (prednisolone 2 mg/kg/day for 4 weeks and then tapered every 4 weeks). The dog was also started on physiotherapy, and the neurological deficits slowly improved over 4 weeks, with full recovery at 8 months. Clinicopathological abnormalities (acute-phase proteins and hypergammaglobulinemia) normalized within 4 weeks.

### Case 2

Despite an otherwise unremarkable physical examination, the neurological findings were characterized by four-limb hypometria, ambulatory tetraparesis without proprioceptive deficits and severely decreased spinal reflexes in all four limbs, compatible with generalized PNS involvement. Therefore, both an inflammatory-immune-mediated/infectious and a neoplastic disease involving the PNS were suspected. The main clinicopathological findings were mild non-regenerative anaemia [hematocrit (Hct) 33% (38–54%)], increased total serum protein [8.2 g/dl (5.7–7.3)], with decreased albumin/globulin ratio [0.41 (0.7–1.30)], globulins [5.8 g/dl (2.8–3.9)], hypergammaglobulinemia [36.9% (6.6–14.5%)], and increased acute-phase proteins [e.g. C-reactive protein [0.56 mg/l (0.01–0.45)] and serum ferritin [606 μg/l (80–270)]. Generalized PNS involvement was confirmed by electrodiagnostic findings with the presence of positive sharp waves on electromyography in the muscles tested (i.e. quadriceps femoris, biceps femoris, tibialis cranialis, biceps brachialis, triceps brachialis, temporalis, longissimus dorsi) and severely decreased motor nerve conduction velocity in both the right peroneal and right ulnar nerves. Based on the electrodiagnostic findings (i.e. denervation to all tested muscles associated with demyelination/axonopathy in all tested nerves), muscle and nerve biopsies were performed.

The histopathological findings in both muscle and nerve (i.e., tibialis cranialis muscle and left peroneal nerve, respectively) were suggestive of inflammatory changes (Fig. 2E, F). Specifically, degenerating fibres surrounded by abundant mononuclear leucocytes and polymorphonuclear neutrophils were present in the muscle specimen, suggesting an inflammatory disease (Fig. 2E); both nerve sections and teased fibre samples showed scattered degenerated fibres surrounded by abundant mononuclear leucocytes, often in areas of focal myelinated fibre loss with occasional collapsed Schwann cell sheaths and concentric collagen proliferation (Fig. 2F). In addition, other large multiple myelinated fibres were embedded by lymphocytes and macrophages. The histopathological findings were consistent with an immune-mediated/autoimmune disease (Fig. 2E, F). Real-time PCR for *Leishmania* spp. on muscle and nerve was positive in all specimens tested. Based on the clinical, clinicopathological, histopathological and parasitological findings, the dog was started on *N*-methyl-glucamine antimoniate (50 mg/kg/twice daily for 4 weeks) and allopurinol (10 mg/kg daily for 6 months) in combination with immunosuppressive corticosteroid therapy (prednisolone 2 mg/kg/day for 4 weeks and then tapered every 4 weeks). The dog slowly improved neurologically within a few days and recovered within 3 weeks. There were no neurological or clinicopathological relapses during the 1-month follow-up. At the time of writing, after 2.5 years, the dog remains in good clinical condition.

## Discussion

The clinicopathological and histopathological findings presented here suggest an association between the active form of CanL and general PNS dysfunction in two dogs. *Leishmania infantum* infection may be responsible for chronic and severe disease in susceptible animals [1], but central and PNS involvement is rarely reported in both humans and dogs as case reports and case series [15–33, 37, 40, 41]. It is recognized that the inflammatory response resulting from the deposition of sCIC in capillary walls with high pressure, turbulent flow and altered vascular permeability is one of the pathogenetic mechanisms during CanL [1, 4, 5, 9]. Therefore, sCIC deposition mainly occurs in specific tissues that are supplied by vessels with the specific characteristics mentioned above [9], as active forms of CanL are often associated with desquamative and ulcerative dermatitis, vasculitis, glomerulonephritis, uveitis and arthritis/polyarthritis [8, 11–14].

**Fig. 3:**
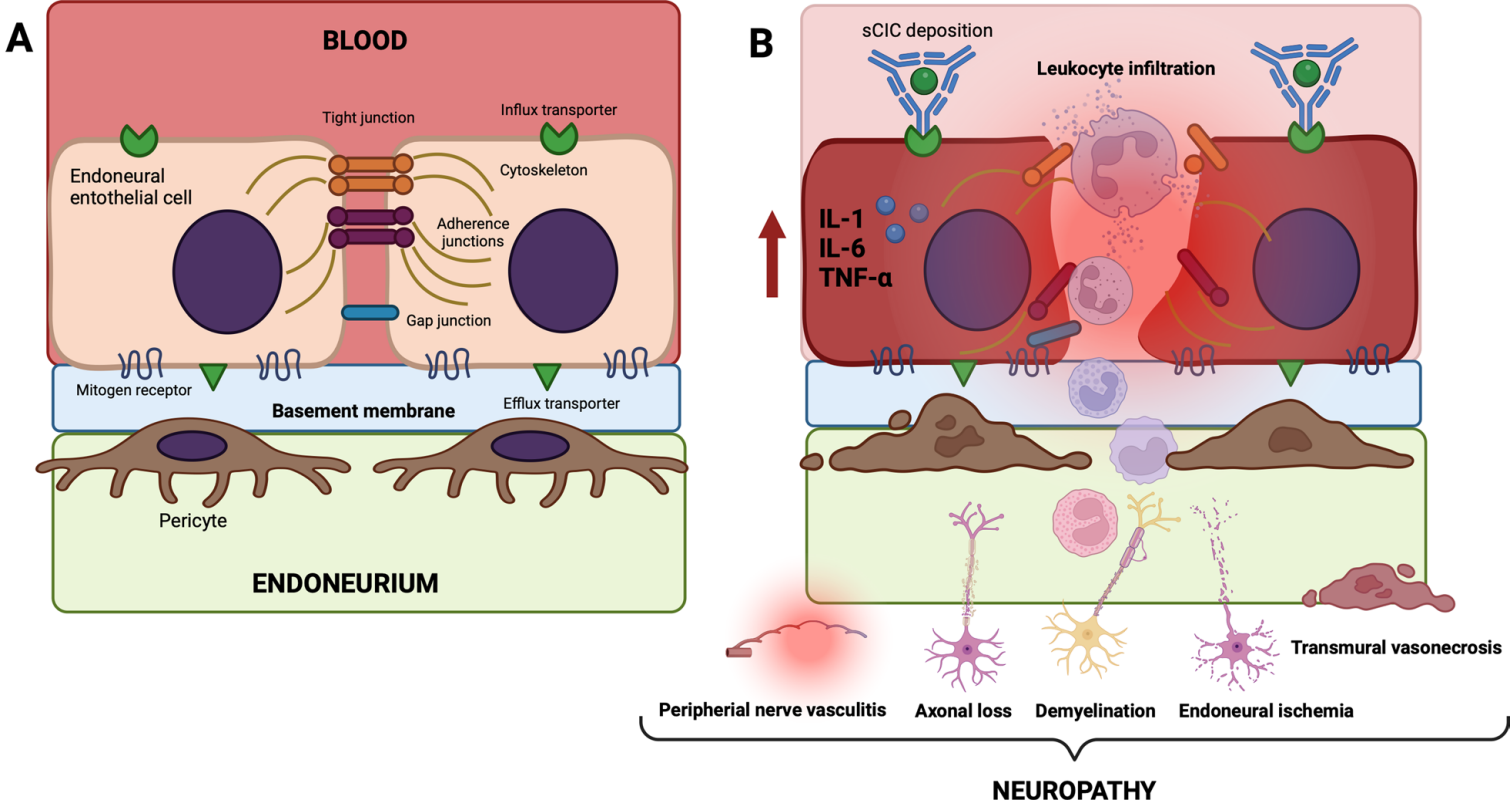
Blood–nerve barrier (BNB) representation (**A**) and BNB breakdown during canine leishmaniosis (**B**). The BNB is located at the innermost layer of the perineurium. The cellular structure of the BNB is formed by endothelial cells, connected by tight junctions, pericytes and the basement membrane. The endothelial cells comprising small endoneurial vessels are non-fenestrated, and the adjacent endothelial cells are connected by complex and continuous tight junctions. The endothelial cells are surrounded by a basement membrane with embedded pericytes (**A**). The soluble circulating immune complex deposition on endoneurial vascular endothelium increases the BNB permeability, contributing to the development of neuropathy. The immune complex deposition is promoted by the activation of transmembrane proteins (i.e. Fc gamma receptors; FcγRs), which induces the recruitment of leukocytes in the peripheral nervous system responsible for peripheral nerve vasculitis, axonal loss and demyelination (**B**)

The pathogenetic mechanism involving the nervous system, both in humans and in dogs, is not well understood, although, as for other tissues, a role of the proinflammatory immune system has been postulated due to an inappropriate and excessive Th2 response against *Leishmania* spp. parasites, more than the presence of the protozoa themselves [15, 34, 35]. As extensively described for the other anatomical regions, the pathogenesis of PNS damage during CanL could be an effect of sCIC deposition on the endoneurial vascular endothelium forming the blood-nerve barrier (BNB) (Fig. 3B). The histopathological results of the nerve biopsies in these two dogs further suggest that a breakdown of the BNB could be the origin of the neuropathy in both cases. This hypothesis is also supported by the histological composition of the BNB (i.e. the endoneurial vascular endothelium and the multilayered ensheathing perineurium), which provides a dynamic, selectively permeable and competent interface between the endoneurial microenvironment and the surrounding extracellular space or blood [42, 43] through the presence of various receptors and transporters on endothelial cells [44] (Fig. 3A).

In addition, the BNB is supplied by slow blood flow and low flow rates to ensure the production of endoneurial fluid by filtration [45], thereby promoting the deposition of sCIC, which may increase the permeability of the BNB and ultimately contribute to the development of the neuropathy (Fig. 3B). While the access of some classes of circulating antibodies (i.e. immunoglobulin A [IgA] and IgM) and haematogenous inflammatory cells (e.g. macrophages) to the BNB is restricted [46], the transport of IgG and immune complexes across the BNB is promoted by the activation of transmembrane proteins (i.e. Fc gamma receptors; FcγRs) [46, 47]. The latter represent the link between the adaptive and innate immune systems, for example during autoimmune diseases [48–50], when FcγRs are downregulated or upregulated [51–53]. Therefore, in the case reports discussed herein, BNB breakdown may have been induced by modulation of FcγRs and sCIC with recruitment of leucocytes into the PNS (Fig. 3B), as confirmed by histopathological findings of nerve biopsies. The above is clearly illustrated by the macrophage and lymphocyte infiltrations associated with inflammatory demyelinating neuropathy findings on histopathology in our dogs. In addition, molecular biological results (real-time PCR) and HIC positivity for *Leishmania* spp. antigen in these two dogs suggest that the pathogenesis of the clinical signs is related to sCIC deposition.

In human medicine, histopathological findings similar to those described here in dogs are suggestive of autoimmune acquired peripheral nerve demyelinating disorders such as acute [i.e. Guillain-Barré syndrome (GBS)] and chronic inflammatory demyelinating polyradiculoneuropathies [54–56]. In these human PNS autoimmune diseases, macrophage infiltration and activating FcγRs are critical elements for endoneurial inflammation mediating axonal injury and multifocal demyelination [54–56], similar to the histopathological findings in our dogs. In most cases, GBS is a post-infectious disease involving the gastrointestinal and respiratory systems [57], and the only infections that have been shown to be significantly associated with GBS are *Campylobacter jejuni*, *Cytomegalovirus*, Epstein-Barr virus, *Mycoplasma pneumoniae*, Zika virus, hepatitis E and *Haemophilus influenzae* [58].

In our dogs, we can assume that the same pathophysiological mechanism as described for GBS occurs in the abnormal immune response induced by *Leishmania* spp. protozoa. However, given that the dorsal root ganglia, nearby spinal roots [42] and neuromuscular junctions [59] are not protected by the BNB [60, 61], the neurological signs could also be a direct consequence of chemotaxis into the PNS, justifying the clinicopathological and histopathological findings in the two dogs. In these circumstances, the addition of an immunosuppressive dose of corticosteroids strengthens our aetiopathogenetic hypothesis of an immune-mediated neuropathy due to CanL. Thus, although an immunosuppressive dose of corticosteroids could be considered inappropriate during a chronic infectious disease such as CanL because of the suppression of adaptive immunity [62, 63], it promotes a marked expression of tight junction proteins [64–66], thereby enhancing the selective permeability of the BNB. All the above is consistent with the established first-line treatment of several inflammatory immune-mediated neuropathies in humans [64], as inferred by the improvement after corticosteroid therapy. Accordingly, treatment with *N*-methyl-glucamine antimoniate alone in case 1 did not guarantee improvement of the clinical signs, and this would support the hypothesis of immune-mediated neuropathy in CanL. In case 2, according to the authors, a combination therapy with *N*-methyl-glucamine antimoniate and steroids at immunosuppressive doses was necessary and justified by the histopathological findings of the nerve biopsy that suggested an immune-mediated disease. Furthermore, other forms of canine immune-mediated polyneuropathy (acute polyradiculoneuritis, chronic demyelinating polyradiculoneuritis, systemic lupus erythematosus, paraneoplastic forms) were excluded by the authors on the basis of clinical signs, laboratory and histology findings and response to therapy [67].

## Conclusions

In conclusion, considering the pathogenesis of organ damage during L.infantum infection as a result of activation of the host Th2 response, the authors suggest that leishmaniasis should be included as a disease able to involve the PNS, especially in endemic areas.

## Data Availability

No datasets were generated or analysed during the current study.
